# CONSTANS-LIKE 5: a key regulator of flower opening and scent emission in *Nicotiana attenuata* and *Petunia axillaris*

**DOI:** 10.1093/plcell/koag025

**Published:** 2026-02-07

**Authors:** Hongwei Jing

**Affiliations:** Assistant Features Editor, *The Plant Cell*,American Society of Plant Biologists, United States; Department of Horticultural Science, North Carolina State University, Raleigh, NC 27695, United States

Flowering is a critical developmental transition in the plant life cycle that determines reproductive success. To ensure successful reproduction, plants precisely regulate flowering time in response to seasonal cues, with photoperiod serving as a major determinant. Day length is perceived by the circadian clock in leaves and relayed to the shoot apical meristem to initiate the floral transition (Maple et al. 2024). In coyote tobacco (*Nicotiana attenuata*), flowers exhibit a tightly regulated nocturnal rhythm of opening, vertical reorientation, and scent emission that optimizes pollination by hawkmoths (Yon et al. 2016). The circadian clock components *LATE ELONGATED HYPOCOTYL* (*LHY*) and *ZEITLUPE* (*ZTL*) play key roles in regulating floral rhythms in *N. attenuata* (Yon et al. 2016). How these circadian signals are translated into coordinated cellular growth and metabolic outputs in flowers has remained largely unknown.

In recent work, **Yuri Choi and colleagues** (Choi et al. 2026) reveal that the transcription factor *CONSTANS-LIKE 5* (*COL5*) functions as a key clock output regulator coordinating flower opening and scent emission in both *N. attenuata* and *Petunia axillaris* (Fig. 1). Building on earlier evidence that *LHY* and *ZTL* regulate floral rhythms, the authors sought to identify downstream transcriptional regulators govering flower opening. RNA-seq transcriptome analysis identified *COL5* as a strong candidate regulator, as it exhibited a diurnal expression pattern and was primarily enriched in the corolla. The time-series expression analysis under both diurnal and continuous dark conditions confirmed that *COL5* transcription is under circadian clock regulation in floral tissues. Furthermore, the authors created *COL5* knockout mutants using a virus-induced gene editing (VIGE) system. These *col5* mutants displayed a consistent phenotype of incomplete flower opening, suggesting that *COL5* is crucial for promoting this process.

**Figure 1:**
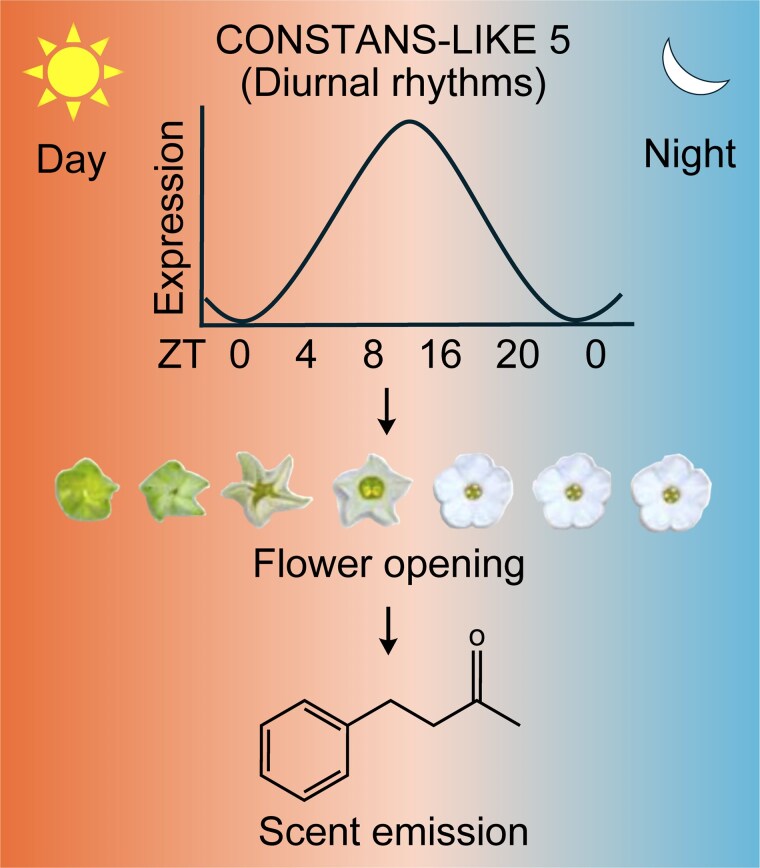
*CONSTANS-LIKE 5* (*COL5*) exhibits diurnal floral rhythms and acts as a key regulator that coordinates flower opening and scent emission in *Nicotiana attenuata and Petunia axillaris*. ZT(0-16): light condition, ZT(16-0): dark condition. ZT, zeitgeber time. Adapted from Choi et al. (2026), Figure 8.

Flower opening is primarily driven by petal cell expansion and/or proliferation (Sun et al. 2021). To test whether *COL5* mediates cell expansion in *N. attenuata*, the authors compared epidermal cell size and number in the corolla limbs. The results showed that *COL5* is required for normal petal cell expansion, thereby promoting flower opening. To further identify downstream targets of *COL5*, the authors performed RNA sequencing on control lines and *col5* mutants. This analysis discovered that transcript levels of genes involved in xyloglucan metabolism and aromatic amino acid biosynthesis were significantly altered in *col5* mutants. In coyote tobacco, the floral volatile benzylacetone is synthesized from L-phenylalanine. Analysis of floral volatiles revealed that benzylacetone was undetectable in *col5* mutants, indicating that *COL5* is required for its biosynthesis. To determine whether COL5's function is conserved across the Solanaceae, the authors examined its ortholog in *P. axillaris*. Phylogenetic and protein sequence analyses confirmed that *COL5* is a conserved ortholog. Consistent with this functional conservation, *PaCOL5* showed circadian-regulated expression under constant dark conditions, and silencing *PaCOL5* reduced the flower opening angle and impaired floral scent emission. Together, these findings demonstrate that COL5's role in coordinating flower opening and scent emission is conserved between *N. attenuata and P. axillaris*.

Overall, Choi et al. (2026) demonstrated that, unlike canonical CONSTANS proteins that control the timing of flowering, COL5 operates after floral induction to coordinate rhythmic floral traits in Solanaceae (Fig. 1). Although their study identifies *COL5* as a central regulator of these processes, several key mechanistic questions remain unresolved. As a member of clade I of the CONSTANS-LIKE family, COL5 contains conserved N-terminal B-box domains and a C-terminal CCT domain, yet the specific functions of these domains in regulating floral development are still unknown. Additionally, while circadian genes such as *LHY* and *ZTL* influence floral opening rhythms, it remains unclear whether *COL5* interacts with these core circadian components. At the cellular level, the molecular mechanism through which *COL5* regulates the expansion of adaxial epidermal cells in the corolla limb has yet to be elucidated. Regarding scent biosynthesis, the molecular mechanism by which *COL5* controls the production of specific volatile compounds, such as benzylacetone, is also unknown. Finally, how the circadian gatekeeping role of COL5 in synchronizing floral opening and volatile emission shapes pollinator behavior in *N. attenuata* and *P. axillaris* remains to be determined.

## Recent related articles in *The Plant Cell*

- Bednarczyk et al. (2025) showed that MADS-box homeotic genes *DEFICIENS* (*PhDEF*) acts as a master regulator of flower development and scent production, both of which are crucial for pollinator attraction in garden petunia (*Petunia* × *hybrida*).
- Skaliter et al. (2023) reported that the R2R3-MYB transcription factor *EPIDERMIS VOLATILE EMISSION REGULATOR* (*EVER*) modulates petunia floral volatile emission by fine-tuning petal epicuticular wax composition, thereby revealing an additional regulatory layer linking wax biosynthesis with floral scent emission in petunia flowers.
- Wu et al. (2025) discovered that the blue light receptor *FLAVIN-BINDING KELCH REPEAT F-BOX 1a* (*ZmFKF1a*) is a circadian-regulated gene that positively controls flowering time, offering important insights into the role of circadian regulation flowering during domestication in maize.
- Zhang et al. (2023) identified *Early heading date 5* (*Ehd5*), a WD40 domain-containing protein that exhibits circadian rhythmic expression pattern and functions as a positive regulator of flowering time, implicating circadian regulation in the control of heading date in rice (*Oryza sativa*).

## Data Availability

No new data were generated or analysed in support of this research.

## Dive Curated Terms

The following phenotypic, genotypic, and functional terms are of significance to the work described in this paper:

COL5 Gramene: A4A49_38205
COL5 Araport: A4A49_38205

PaCOL5 Gramene: Peaxi162Scf00942g00122
PaCOL5 Araport: Peaxi162Scf00942g00122

## References

[koag025-B1] Bednarczyk D et al 2025. The homeotic gene PhDEF regulates production of volatiles in petunia flowers by activating EOBI and EOBII. Plant Cell. 37:koaf027. 10.1093/plcell/koaf027. PMC11850304 39913239

[koag025-B2] Choi Y et al 2026. *CONSTANS-LIKE 5* facilitates flower opening and scent biosynthesis in Solanaceae. Plant Cell. 38:1–13. 10.1093/plcell/koag016. 41605244

[koag025-B3] Maple R, Zhu P, Hepworth J, Wang JW, Dean C. 2024. Flowering time: from physiology, through genetics to mechanism. Plant Physiol. 195:190–212. 10.1093/plphys/kiae109. 38417841 PMC11060688

[koag025-B4] Skaliter O et al 2023. The R2R3-MYB transcription factor EVER controls the emission of petunia floral volatiles by regulating epicuticular wax biosynthesis in the petal epidermis. Plant Cell. 36:174–193. 10.1093/plcell/koad251. 37818992 PMC10734618

[koag025-B5] Sun X et al 2021. Molecular understanding of postharvest flower opening and senescence. Mol Hortic. 1:7. 10.1186/s43897-021-00015-8. 37789453 PMC10514961

[koag025-B6] Wu F et al 2025. The blue light receptor ZmFKF1a recruits ZmGI1 to the nucleus to accelerate shoot apex development and flowering in maize. Plant Cell. 37:koaf199. 10.1093/plcell/koaf199. PMC12449209 40811583

[koag025-B7] Yon F et al 2016. Silencing Nicotiana attenuata LHY and ZTL alters circadian rhythms in flowers. New Phytol. 209:1058–1066. 10.1111/nph.13681. 26439540 PMC5147715

[koag025-B8] Zhang X et al 2023. The WD40 domain-containing protein Ehd5 positively regulates flowering in rice (Oryza sativa). Plant Cell. 35:4002–4019. 10.1093/plcell/koad223. 37648256 PMC10615205

